# Analysis of Tube-to-Tubesheet Welding in Carbon Steel Heat Exchangers of a Double Plate Header Box

**DOI:** 10.3390/ma15010261

**Published:** 2021-12-30

**Authors:** José García González, Juan José Hernández-Ortega, Ana-Eva Jiménez-Ballesta, Rosendo Zamora Pedreño

**Affiliations:** 1 Mecánicas Bolea S.A., 30353 Cartagena, Spain; jgonzalezga@hotmail.com; 2 Departamento de Ingeniería de Materiales y Fabricación, Universidad Politécnica de Cartagena, 30202 Cartagena, Spain; anaeva.jimenez@upct.es (A.-E.J.-B.); rosendo.zamora@upct.es (R.Z.P.)

**Keywords:** tube-to-tubesheet welding, double plate header box, orbital GTAW, parallelism deviations, air-cooled heat exchanger

## Abstract

The rear wall of the header box serves as a tubesheet in heat exchangers of double plate header box. Tube-to-tubesheet welding must be performed using orbital Gas Tungsten Arc Welding (GTAW) with a head extension, which is passed through the corresponding hole in the front wall (plugsheet) of the header box, where the welding machine is supported. In this project, the effect of parallelism deviations between the plugsheet and the tubesheet of carbon steel header box is analyzed to evaluate its influence on the quality of the tube-to-tubesheet welding. Welded tube (SA-210 Gr. A1) to tubesheet (SA-516 Gr. 70) coupons are manufactured simulating the parallelism deviations previously analyzed in two double plate header boxes of air-cooled heat exchangers using two different preheating temperatures. Macrographic analysis is performed in order to evaluate the weld penetration (minimum leak path) and length of the weld leg in tube-to-tubesheet joints. The results obtained show important variations in those parameters when the parallelism deviations are equal to or greater than −1 mm over the theoretical distance as well as when the distance approaches +1 mm or more. Finally, the incorporation of dimensional controls prior to the welding process is discussed and the implementation of improvements in orbital GTAW equipment is recommended as an optimal solution for this kind of heat exchangers.

## 1. Introduction

Heat exchangers are a key component in several industries. They can be found in petrochemical plants; chemical and pharmaceutical industries; steam power plants; nuclear reactors; and water power plants amongst other industries [1]. Heat exchangers are basically used to transfer heat from one medium to another, in both cooling and heating processes. Depending on the process, liquids, gases or both must be heated, evaporated, cooled, or condensed. The requirements that heat exchangers must fulfill as well as their design, depend on the individual operating area. For example, in chemical or petrochemical plants they can be in contact with aggressive agents, in combination with elevated temperatures and high pressure. In other industries, such as the pharmaceutical industry or food processing, the requirements can be very different, and the contamination of the fluids can be critical [1]. One of the greatest challenges with heat exchangers is to achieve a high reliability during service because they are exposed to important variations in operational parameters and severe conditions related to the fluids they are in contact with. Ali et al. [2] conducted a review of the most common modes and causes of failure in heat exchangers. Fouling, scaling, salt deposition, weld defects and vibrations are among the most common causes of such failures and usually appear in the form of cracks and leaks due to the effect of the stresses generated in the components.

**Figure 1:**
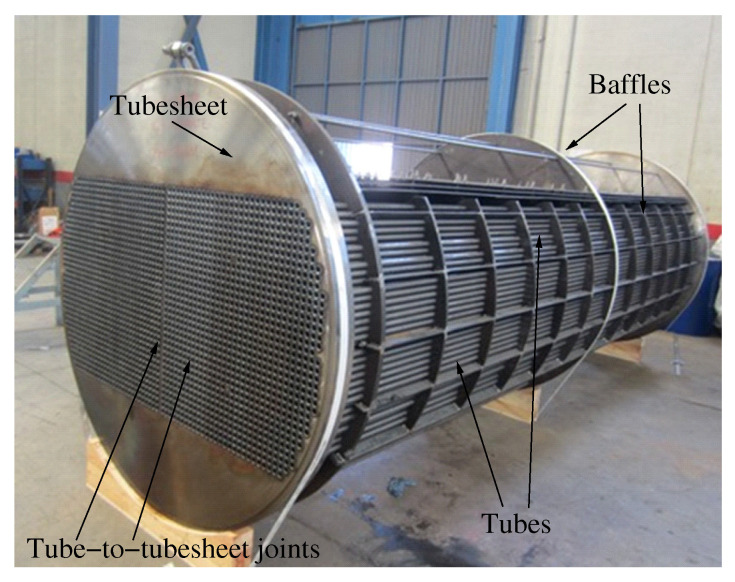
Photograph of some components of a shell and tube heat exchanger.

One of the most critical elements in many types of heat exchangers is the joint between the tube and the tubesheet [3,4]. Figure 1 shows a photograph of some components of a shell-and-tube heat exchanger: tubesheets, tubes and their joints.

The design of tube-to-tubesheet joints varies widely depending on the severity of the service conditions [5]. They can be made by tube expansion, by welding, or by a combination of welding and plastic deformation.

For welded joints, several types of recommended configurations (structures) of the joint can be found in [6]. Wei and Ling [7] experimentally assessed the effects of different welded configurations on mechanical properties of tube-to-tubesheet joints. Farrahi et al. [8] also examined two types of tube-to-tubesheet welded joints in heat exchangers of a petrochemical unit. In this case, they employed the finite element method to simulate the welding process and post weld heat treatment (PWHT) in order to find the factors affecting the failure in tube-to-tubesheet welds. Three approaches for designing the configurations of the tube-to-tubesheet joints in steam generators of nuclear power plants, including material and manufacturing were analysed in [4]. Another important point in this kind of equipment is the performance of dissimilar joints [9,10], which can be necessary in harsh environments in order to minimize the overall operating cost.

Many failures in tube-to-tubesheet joints are repaired by plugging the tubes and thereby leaving those tubes totally out of service, although Farrahi et al. [11] concluded that plugging the tubes should only be considered as a temporary solution.

Weld defects during the manufacturing stage can be the cause of many failures in service, due to the presence of porosity, inclusions, lack of fusion and penetration, shrinkage cracks or internal stresses [12]. Therefore, the selection of appropriate base and weld material, process parameters, performance of the welding, application of heat treatments and release of mechanical stresses are well known factors that generally speaking must be taken into account [5,13].

Otegui et al. [5] analyzed and discussed the causes of multiple cracks found in a heat exchanger tubesheet at a petrochemical plant, which had been repaired several times. The analysis they performed showed embrittlement of the heat affected zone (HAZ) of the welds, and a lack of penetration and fusion at the weld roots. These nonconformities in the weld were pointed to as being the causes of the first cracks.

In a previous analysis made by the same authors regarding crack propagation in five gas-steam heat exchangers, they identified a mechanism of microstructural grain-boundary embrittlement. In that case, high residual stresses in tube-to-tubesheet welds, and high in-service applied mechanical stresses contributed to the damage [13].

In the same way, Liu et al. [12] studied failures in tube-to-tubesheet welded joints of a shell-tube heat exchanger to determined its failure mechanism. A combination of welding and an expansion process after the welding was used for the joint between tube (AISI 304 stainless steel) and the tubesheet (SA 516 Gr. 70). That analysis suggested that serious defects found at the welded joint were the origin of the cracks and also probably an unsuitable expansion position of the tube after welding, which contributed, together with residual stresses, to the formation of the initial cracks.

More failures have been investigated with relation to heat exchangers and particularly with tube-to-tubesheet joints [14,15,16,17,18], but the causes were related to operating conditions in most cases: fluid-induced vibration [14]; joint fatigue strength [15]; chloride accumulation [16]; carbon pick-up; and an overload mechanism during a maintenance shut down [17]. The root cause was related to a lack of fusion in the weld joints in just one study [18]. However, weld joints of tube-to-tubesheet are always mentioned as the zone of crack propagation.

The large amount of tubes of this kind of equipment [4,14] has a major impact on the manufacturing cost [19], so it is necessary to increase the quality of the welds and the productivity of the welding process. Orbital Gas Tungsten Arc Welding (GTAW) or Tungsten Inert Gas (TIG) is the most widely used process for this kind of joints, although other processes have also been studied [19].

Recently, Lei et al. [20,21] conducted two interesting reviews. The first one was vision-aided robotic welding [20] and, the second one was the development of the automation and digitization of the tube-to-tubesheet welding [21]. As they pointed in [21], the positioning of the welding gun is one of the key technologies in guaranteeing the welding quality, and it mainly includes tube center positioning and welding height positioning. In their work [21], the available technologies to achieve the correct tube center positions, welding height positioning, and arc length control were well described. A more detailed description of some of these technologies can be found in [22] for a welding path detection using passive vision, and [23] for welding control height using a cross-lines laser aided machine vision.

**Figure 2:**
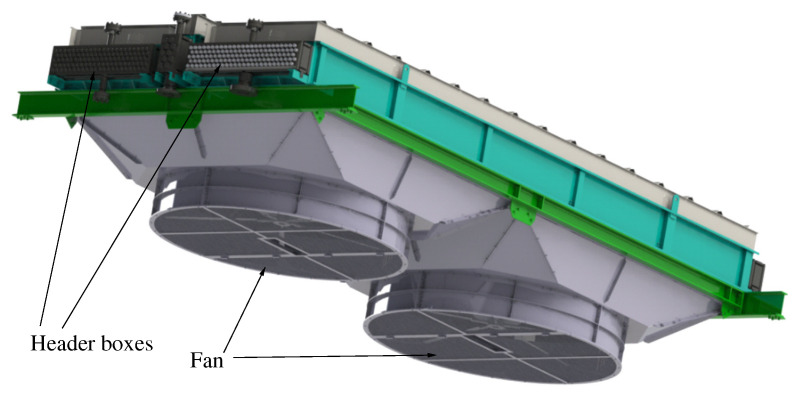
Part of an air-cooled heat exchanger.

In general, a wide variety of heat exchangers can be found in the industry [2]. Although the most common type of heat exchanger used in the oil and petrochemical industries is the shell and tube heat exchanger (Figure 1) [24], a challenging task in the field of mechanized welding is to perform tube-to-tubesheet welds in the closed header box of an air-cooled heat exchanger [1], which is what this work focuses on. Figure 2 shows part of an air-cooled heat exchanger, containing four header boxes. In the image, two of the four header boxes have been identified.

**Figure 3:**
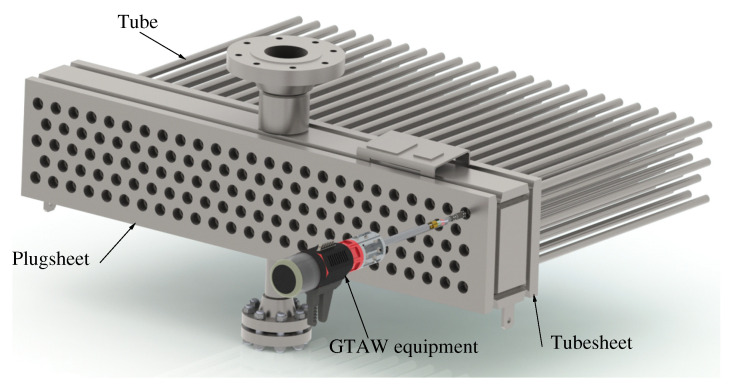
One header box of a heat exchanger with five rows of tubes and the orbital GTAW equipment positioned for operation.

The header boxes studied in this project are manufactured from six carbon steel plates welded together: front plate (plugsheet); rear plate (tubesheet); top and bottom plates and two end plates completing the box construction. There are also internal plates for structural integrity. The plugsheet has the same number of holes as the tubesheet but they are threaded. During operation the plugsheet holes are sealed using gaskets and removable plugs. These plugholes are the only access for the welding equipment to join the tubes to the tubesheet during manufacturing as the header box is completely constructed before the tubes are joined (Figure 3). The plugholes are also used for joint inspection after fabrication of the heat exchanger.

**Figure 4:**
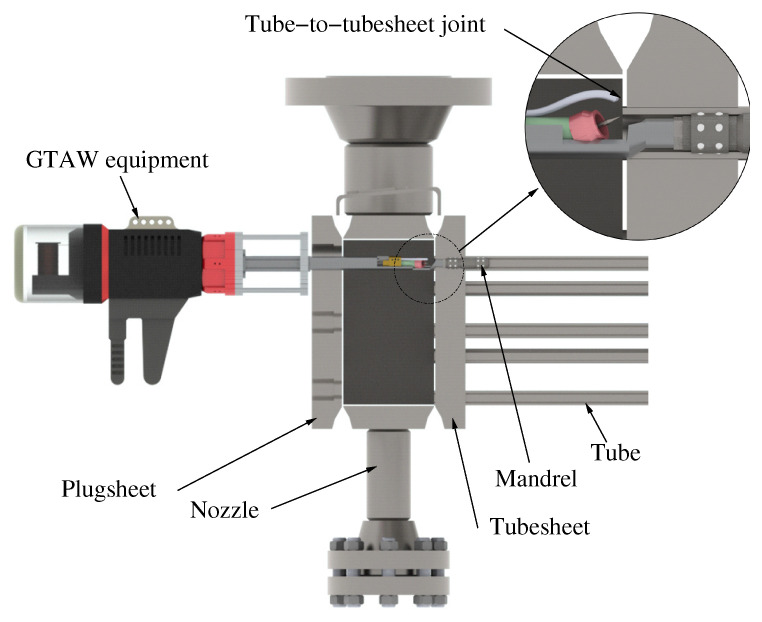
Detail of how orbital GTAW equipment accesses the tube-to-tubesheet joint to carry out the welding.

In this kind of heat exchangers, tube-to-tubesheet welding is usually performed with an orbital TIG machine passing through the closed header box (Figure 4). The orbital tube-to-tubesheet welding head is equipped with an adapter, which is passed through the corresponding hole in the plugsheet. A centering mandrel is introduced into the tube and the welding is performed.

Studies can be found about this kind of heat exchanger in [25,26,27,28], but they are related to the design and analysis of the header box, especially the nozzle joints.

There are two issues that are difficult to properly prevent in this operation during the actual manufacturing practice performed in industry. The first difficulty is to achieve correct positioning of the orbital TIG equipment [1], as that has to be done through a closed head with no visibility for the weld operator (Figure 3 and Figure 4). A mandrel introduced into the tube centres the equipment with respect to the actual joint. The rear wall of the header box is used as a tubesheet, where the tube ends are inserted into the designated holes. After introducing the welding lance, the operator proceeds to make the calibration of its position: the distance between the electrode tip and the joint is fixed manually using the front wall as a reference, but with no visibility of the rear wall where the joint has to be made. Then, the distance between the electrode tip and the joint is held for the whole header box, due to the difficulty of the operation and the number of tube-to-tubesheet welds to be made in each head, for productivity reasons.

The second difficulty is related to preheating the tubesheet and tubes to be welded in the workshop; this operation is highly complicated due to the dimensions, configuration and geometry of the header box.

The aim of the present work is to focus on the study of the consequences of keeping a fixed position distance for the orbital TIG machine on the weld quality of the tube-to-tubesheet joints, which corresponds with the first issue, when deformation in the header box is produced. However, preheating temperature influence is also taken into account in the analysis of the weld quality.

In our own previous experience, leaks were detected in a heat exchanger of a double plate header box after one year of service due to transverse cracks in the tube-to-tubesheet welds. The failure hypothesis was that a lack of penetration due to deficient positioning of the TIG equipment would have contributed to inadequate joint resistance, stress concentration and a crack formation. The hypothesis about a deficient positioning of the TIG equipment was based on the fact that the manufacturing process of the header box could provoke deformations in the box and a lack of parallelism between the front and rear walls. Thus, if the welding operator maintained the initial calibration of the equipment, it would have produced differences in the length of the welding arc and, consequently, a lack of penetration and low quality in the tube-to-tubesheet weld. Performing repetitive calibrations of the welding lance position is complex and expensive to carry out in industrial practice.

In the current work, the maximum parallelism deviation of double plate header boxes for the petrochemical industry is quantified and its influence on the quality of the welded tube-to-tubesheet joints is evaluated; practical solutions are also proposed. The quality of the joints will be evaluated quantitatively in terms of penetration of the weld (minimum leak path “MLP” and length of the leg $A_{f}$) as well as qualitatively, with the shape of the weld.

To the best of the authors’ knowledge, this is the first research that focuses on the effects of header box distortion over tube-to-tubesheet welds in this kind of heat exchanger.

In order to achieve our objective, we have built two header boxes and carried out their metrological control in terms of parallelism deviation. Subsequently, two coupons or mockups have been designed and welded simulating these measured deviations in the welding process of tube-to-tubesheet joints. All the welded joints of the mockups where made according to a standard procedure for the petrochemical industry [6], using two preheating temperatures for each case.

## 2. Materials and Methods

This section initially describes the materials and procedures used to manufacture two header boxes that are part of an aero-cooled heat exchanger used in the petrochemical industry and the dimensional control carried out to quantify the parallelism deviation between the two main plates of the head. Subsequently, a mockup (coupon) design is detailed that reproduces the parallelism deviations found between the plates and the procedure used to weld the tube-to-tubesheet joints. Finally, the methods for extracting and preparing the specimens from the coupon, and how to measure and analyze the quality of tube-to-tubesheet joints are explained.

### 2.1. Manufacturing of Two Models of Header Box

**Figure 5:**
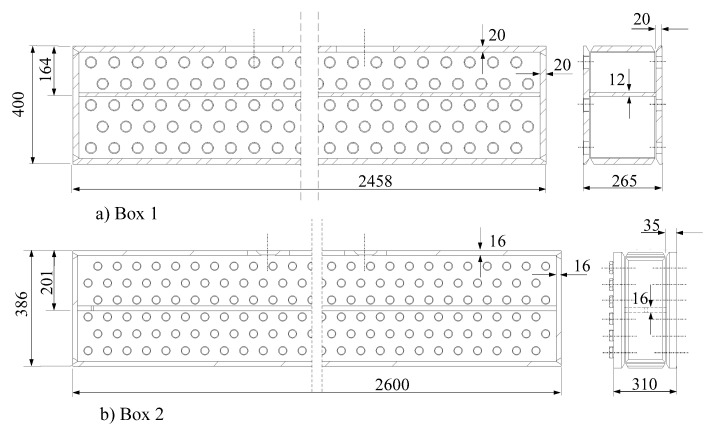
Dimensions (in mm) of header boxes.

The two header boxes manufactured to evaluate parallelism deviations correspond to the dimensions indicated in (Figure 5). The material of the header boxes is SA-516 Gr. 70 (Table 1). The dimension and tubesheet thickness differed in each header box. They were manufactured following qualified welding procedures by a company that is specialized in these type of equipment and which has vast experience.

It is well known that welding causes deformations in large parts. Strategies such as appropriate selection of the welding sequence, heat distribution, and tack welds were used to minimize deformations during the manufacturing of the header boxes. In this case, a stiffening sheet, provisional cylindrical bars and provisional plates were also employed to keep the plates level and aligned during the welding.

Once the box had been welded, two holes were made to place and weld the head nozzles. After that, a post-welding heat treatment was carried out to relieve stresses at 595 ± 15 °C at 1 h/25.4 mm.

Next, holes were machined in the plugsheet and tubesheet of the exchanger by a numerical control milling machine. In our case, one of the boxes had five rows of holes (Box 1), whilst the other one had six (Box 2).

Finally, the two header boxes were taken to the metrology laboratory. A metrological control of the two header boxes was carried out using a three-coordinate Trimek measuring station to measure the parallelism variations between the plugsheet and the tubesheet. It should be stated that this metrological control is not a typical stage in the manufacturing process for this kind of equipment.

### 2.2. Mockup Manufacturing

**Figure 6:**
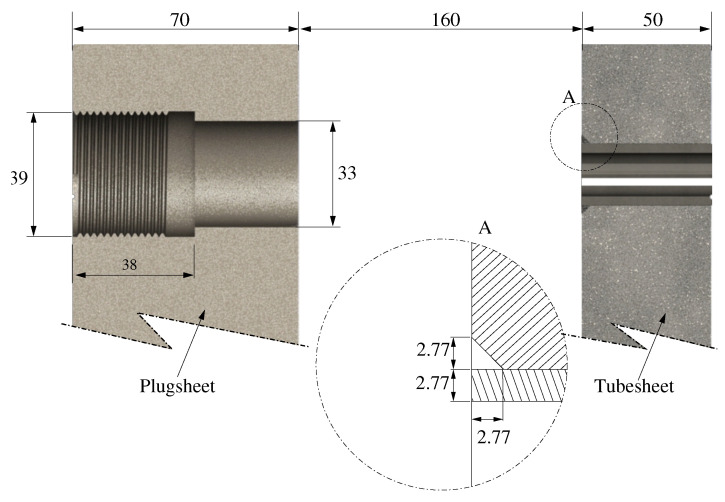
Disposition of plates (plugsheet and tubesheet) in the mockup. Detail A: design of the tube-to-tubesheet joint. (All measurements are in mm).

The mockup was designed and manufactured to evaluate the effect of the parallelism deviations on the tube-to-tubesheet weld, and consisted of two plates of 70 and 50 mm thickness (Figure 6). The plates were joined and kept at a fixed distance of 160 mm using cylindrical bars with tack welds on both plates. One of the plates (70 mm thick) functioned as the plugsheet (front wall of the header box), and it was machined to make the holes through which the orbital TIG equipment could access the tube-to-tubesheet joint. The other plate (tubesheet) was 50 mm thick, which is the standardized thickness according to ASME [30] for the qualification of the welding procedure. This plate (rear wall of the header box or tubesheet) contained the holes where the exchanger tubes were inserted.

**Figure 7:**
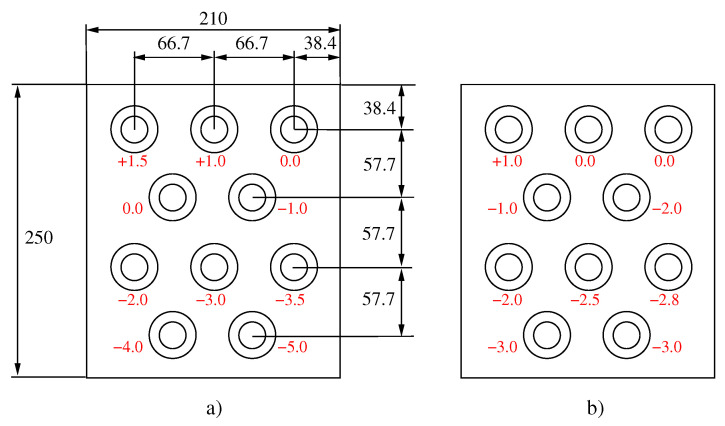
Layout of coupons designed for evaluating parallelism deviation (in red color close to each hole) (**a**) Mockup 1 (100 °C preheating); (**b**) Mockup 2 (200 °C preheating). (All measurements are in mm).

The mockups had four rows of holes with the layout that is shown in Figure 7. On the 70 mm plate, the support holes (plugs) of the orbital TIG equipment were threaded because they needed to support the TIG equipment.

The surface of the tubesheet was machined with different depths in the area around the holes. These machinings reproduced the parallelism deviations found by the previous metrological control of the header boxes. In the mockup, these deviations ranged from +1.5 mm to −5.0 mm for the first coupon and +1.0 mm and −3.0 mm for the second coupon. In the mockup, negative deviations represented distances between the electrode-joint larger than that fixed during calibration, and positive deviations represented shorter distances. The selection of these values is discussed in Section 3.1. The depths were indicated close to each hole in Figure 7, with the distance 0 representing the theoretical reference position of the orbital TIG equipment in the calibration and the negative and positive values referred to greater distances or lesser distances, respectively, between the electrode and the joint. The machining of the two plates of each mockup was carried out so that the holes of both plates were concentric.

#### Welding Specification Procedure

The preparation of edges corresponding to the detail (b) of the ASME BPVC VIII-1 2019 (Figure 6).

The material of the plates and the tubes were SA-516 Gr. 70 and SA-210 Gr. A1, respectively (Table 1). The tubes were made of SA-210 Gr. A1 according to [29]. Tube dimensions were 25.4 mm in diameter and 2.77 mm in thickness. The carbon equivalent content is also shown in Table 1.

The preheating temperature of thick plates is also a critical parameter in the welding procedure specification (WPS). In the case of heat exchangers, preheating is extremely complicated to perform because of the considerable dimension of the tubesheet and the significant number of tubes. Although the metallurgist aspect of the welding is not the aim of this study, two preheating temperatures were considered in order to analyze their influence on the weld penetration. The first coupon was welded with a preheating temperature of 100 °C and the second one with 200 °C. This latter is the temperature indicated in the welding procedure. The temperature of 100 °C was introduced to assess how a decrease in the preheating temperature affected the weld penetration. Both coupons were welded with two runs, the first run was without filler material (142 process according to [31]) and the second one (141 process according to [31]) with filler material AWS A5.18 ER-70S-6 [32] and a diameter of 0.8 mm. A direct current straight polarity of square wave was used. The base welding current was 10 A in the first run, and the peak welding current was 240 A. The pulses were of 0.20 s. The welding speed was 1.3 rpm/min. In the second run, the peak welding current was 220 A with 0.20 s, while the base current was 40 A with a duration of 0.40 s. A mixture of Argon and Helium, SFA 5.32 SG-AHe-25 [32] (ISO 14175 I3-ArHe-25 [33]) was used as a protective gas with a flow rate of 20 l/min during the root run. Argon, SFA 5.32 SG-A [32] (ISO 14175 I1-Ar [33]) was used as a protective gas with a flow rate of 10 l/min during the second run. In the calibration, the electrode-part distance was 2 mm, therefore that distance was used as the reference (case of 0 mm of parallelism deviation in the mockup).

**Figure 8:**
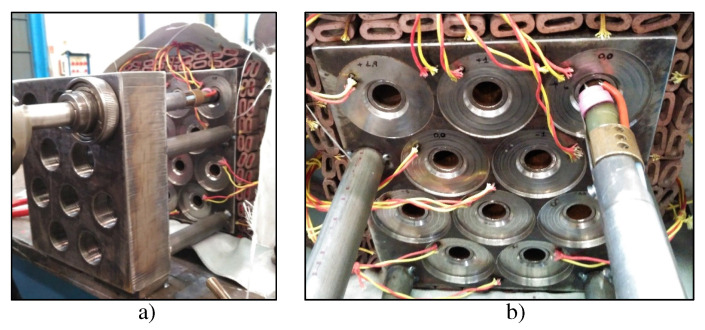
Mounting and welding of mockup 1 during the test: (**a**) General view; (**b**) closer view of tube-to-tubesheet joint with machined holes (parallelism deviations).

In Figure 8 the mockup can be seen with the TIG equipment positioned and prepared for welding. The machined surface around the different holes can also be observed. To begin welding the coupon, the welding operator positioned and calibrated the electrode-part distance in the TIG equipment for the referenced joint named as a deviation of 0 mm. The rest of the joints were welded without the operator modifying that regulation. Thus, that electrode distance piece varied depending on the deviations introduced in the coupon, as in an actual header box.

The first run without filler material was completed without problems at all joints where the deviations were negative, i.e., with distances between the electrode-joint exceeding 2 mm (calibration position). However, visual inspection of that first pass showed poor welding in both the −4 mm and −5 mm deviations. In the case of positive deviations (distances between the electrode-joint of less than 2 mm), there were weld problems with the deviation +1.5 mm, so it was incomplete, and its performance was discarded.

The second run with filler material was not completed in the joints with a negative deviation higher than −3.5 mm because the filler metal dropped and adhered to the electrode.

### 2.3. Macrographical Analysis

**Figure 9:**
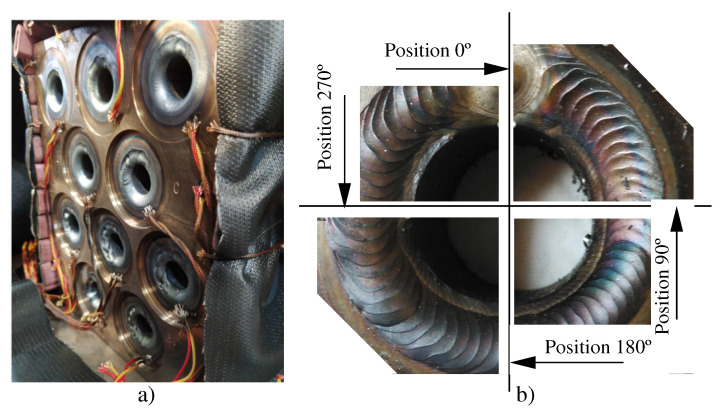
Tubesheet of mockup 2 after finishing the welds. (**a**) General view; (**b**) Positions of coupon cut in a joint to obtain the specimens.

Once the welds had been made in the mockups, each tube-to-tubesheet joint was cut into four pieces as shown in Figure 9. The cuts were identified with the positions 0°, 90°, 180° and 270°. Specimens were extracted from these parts by lubricated cutting with linear precision saw ISOMET 4000 (Buehler USA, Lake Bluff, IL, USA). After that, all cut specimens were hot mounted in 40 mm diameter samples, using XPHB phenolic resin. For optimal macrographical observation and measurement, they were grinded with XPNC SiC abrasive papers to a maximum P2400 grit, and then polished with XALO alumina suspension of 1 and 0.3 µm.

Nital 3% solution (3% HNO${}_{3}$ in ethanol) was used to reveal the weld and the HAZ. The measurement of the minimum leakage path (MLP) and length of the weld leg ($A_{f}$) in each sample was carried out with LAS v4.13 software (Leica Microsystems). Macrographs were taken with a Leica DMRX optical microscope equipped with a Leica MC190 HD camera. For each parallelism deviation value tested, four metallographic samples were obtained and measured (one of each quadrant of the orbital weld).

## 3. Results and Discussion

In this section, the parallelism deviation between the plates of the header box is shown and analyzed. Then, the weld penetration in the tube-to-tubesheet was studied, evaluating the influence of the parallelism deviation and the preheating temperature over the weld quality.

### 3.1. Parallelism Deviation

**Figure 10:**
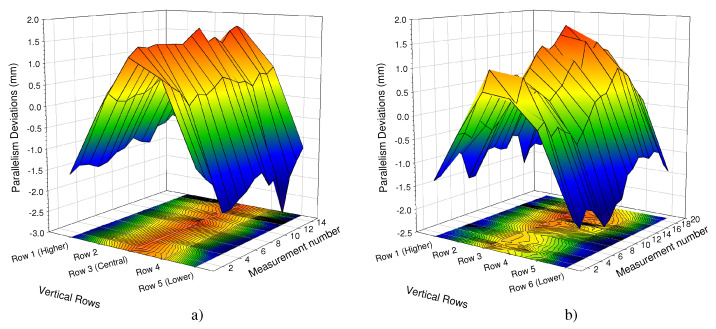
3D representation of parallelism deviations of: (**a**) Box 1; (**b**) Box 2.

**Figure 11:**
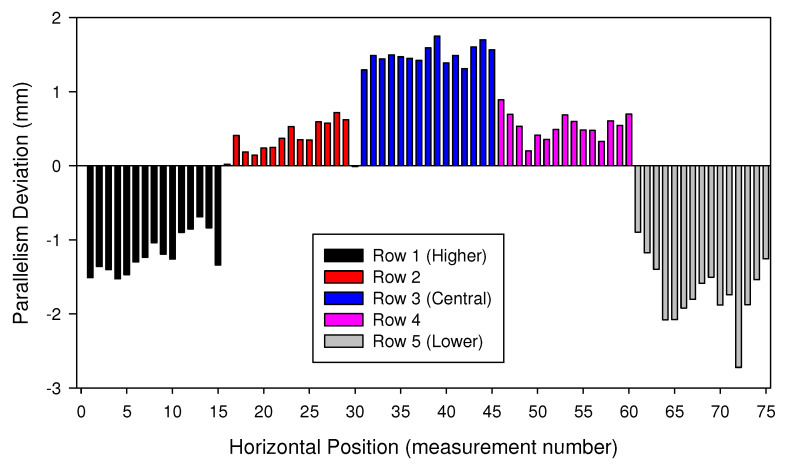
Deviations measured in Box 1 for each row of holes. Average measured distance is taken as the reference (zero value).

**Figure 12:**
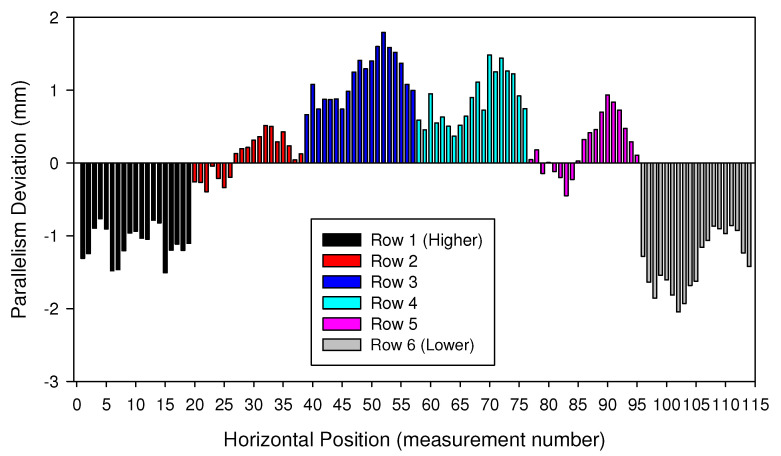
Deviations measured in Box 2 for each row of holes. Average measured distance is taken as the reference (zero value).

Figure 10, Figure 11 and Figure 12 show the parallelism deviations between the plugsheet and tubesheet along the length of the box for each of the five rows that header box 1 consisted of and the six rows that header box 2 consisted of, measured by Trimek equipment.

In both cases (Figure 11 and Figure 12), it can be observed how the distance between the plugsheet and tubesheet in the higher and lower rows of the header boxes is less than the average distance between planes. In these figures, a negative value represented a shorter distance between the plates than the average distance. On the contrary, the distance in central rows was greater than the average distance between plates. This shortening at the ends is logical because the solidification and cooling of the longitudinal weld of the plates produces a contraction. It can also be observed that the most significant distances in the central rows occurred in the middle of the plates.

The maximum deviations in both boxes were −2.75 mm and 1.75 mm, depending on the zone of the box.

In the most unfavorable case, the maximum errors that could occur would be the sum, in absolute values, of both values (−2.75 mm and 1.75 mm), depending on the zone used to calibrate it. Thus, if the same calibration is maintained throughout the welding of all the holes without performing any intermediate control or check, significant deviations could appear.

For that reason these maximum values were taken as a reference to design the mockup of the tube-to-tubesheet joint described in the previous section, ranging from −5 mm to 1.5 mm in 0.5 mm steps.

The deviations between plugsheet and tube sheet are due to the manufacturing procedure of the header box. Welding processes and PWHT provoke deformations in the box and, therefore, a lack of parallelism between the front and rear walls. The value and distribution of those deviations depend on the dimensions of the header box, welding sequence, heat distribution, stiffening sheets among other parameters, and are very difficult to avoid and control.

### 3.2. Weld Penetration

**Figure 13:**
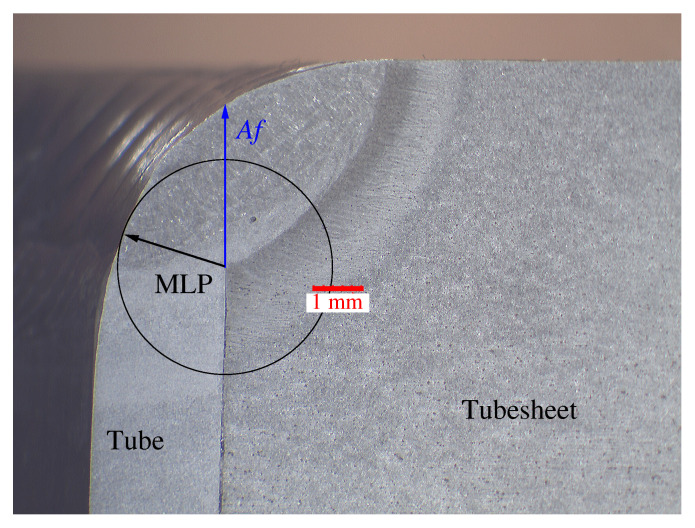
MLP and $A_{f}$ in a macrograph of the tube-to-tubesheet joint.

This section discusses the influence that parallelism deviations have on the weld penetration of the tube-to-tubesheet joint. Moreover, some consideration is given to the influence of the preheating temperature on the weld penetration. The penetration of the joint is measured according to standards on the macrograph of each specimen (Figure 13). The parameters measured were: according to [34] the minimum leakage path (MLP), and according to ASME BPVC VIII Div 1 UW-20.6 [6] the length of the weld leg ($A_{f}$) (Figure 13). For full strength welds, the MLP must be at least equal to 90% of the thickness of the tube (0.9 × 2.77 = 2.493 mm) and the minimum required $A_{f}$ must be greater than 2.70 mm.

**Figure 14:**
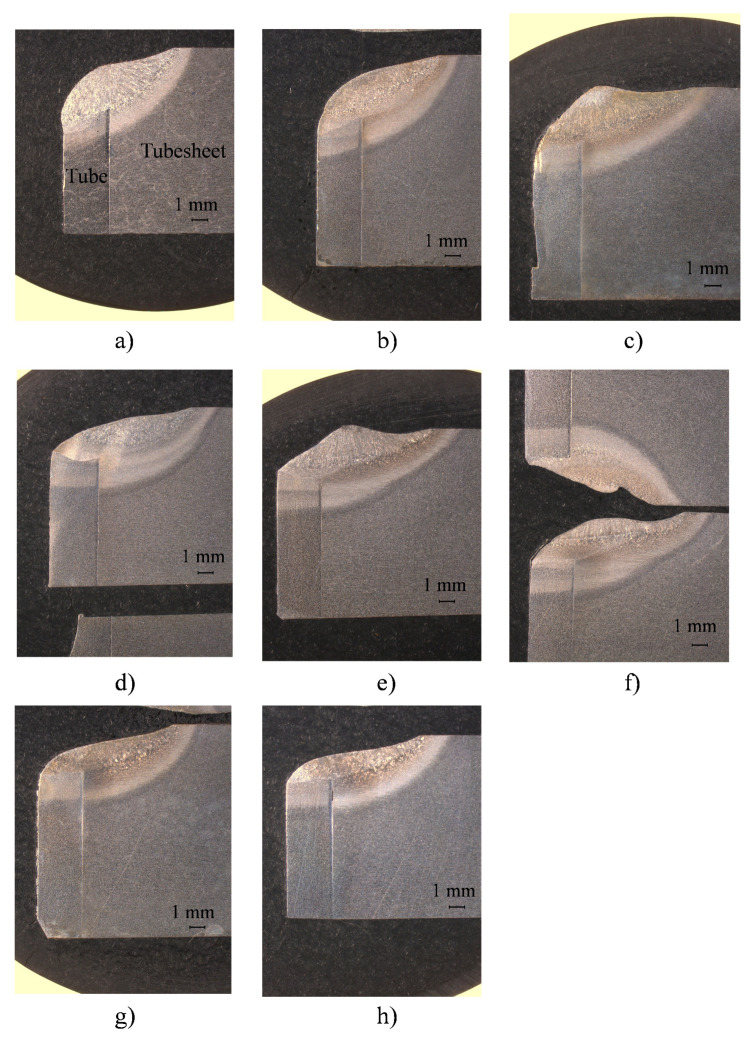
Macrographs of specimens at position 0° cut. Deviations in specimens of mockup 1: (**a**) +1 mm; (**b**) 0 mm (calibration value); (**c**) −1 mm; (**d**) −2 mm; (**e**) −3 mm; (**f**) −3.5 mm; (**g**) −4 mm and (**h**) −5 mm. In all macrographs, tube is on the (**left**) side and tubesheet on the (**right**) side.

**Figure 15:**
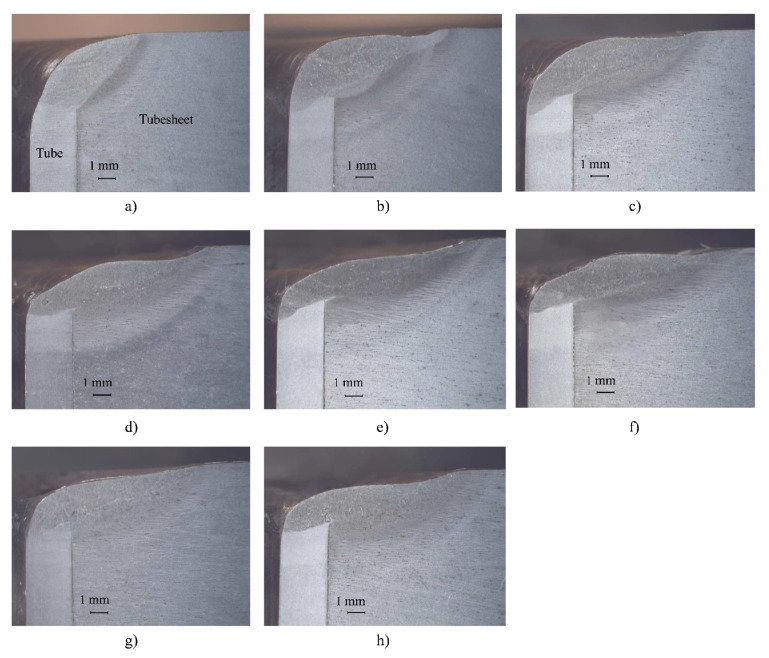
Macrographs of specimens at position 0° cut. Deviations in specimens of mockup 2: (**a**) +1 mm; (**b**) 0 mm (calibration value); (**c**) −1 mm; (**d**) −2 mm; (**e**) −2.5 mm; (**f**) −2.8 mm; (**g**) −3 mm and (**h**) −3 mm. In all macrographs, tube is on the (**left**) side and tubesheet on the (**right**) side.

Figure 14 and Figure 15 show the macrographs obtained from the cuts made to the tube-to-tubesheet joints of coupon 1 and coupon 2, respectively, in position 0°. It can be seen that the shape of the weld (fusion zone and HAZ) changes as a function of the parallelism deviations introduced in the mockup. For some parallelism deviations, the shape of the weld is very irregular and deviates from the shape of the weld obtained for the calibration distance value (0 mm of parallelism deviation, Figure 14b and Figure 15b).

**Figure 16:**
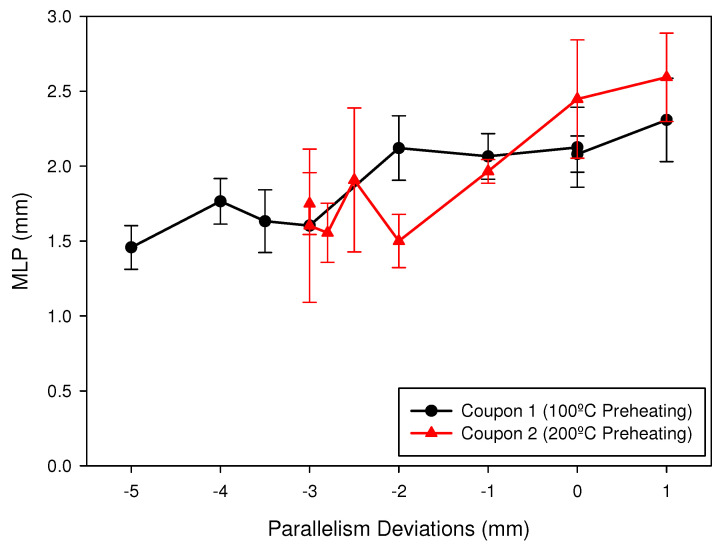
Minimum Leakage Path (MLP) measured for the analyzed coupons.

Figure 16 shows the variation of the minimum leakage path (MLP) as a function of the parallelism deviation induced in the coupons (electrode-part distance) for the two coupons analyzed. The arithmetic mean of the MLP was obtained from the four analyzed positions (0°, 90°, 180° and 270°) and the standard deviation has been represented at each point. The black line in Figure 16 represents MLP for the welding made with a preheating temperature of 100 °C, and the red line represents the MLP for the welding made with a preheating temperature of 200 °C. It can be observed that the MLP decreased in both coupons with the increase, in absolute value, of the negative parallelism deviation (greater distance between electrode and joint). For deviations greater than −2 mm, in absolute values, the MLP was always less than 2 mm. It was also observed that for coupon 2, a higher MLP was obtained for deviations 0 and 1 mm. In the case of 200 °C of preheating, the arithmetic mean of the MLP value was sufficient to meet the standard (2.493 mm in our case) in values of parallelism deviation around the optimal distance. However, in the case of coupon 1, the MLP value was not acceptable even for the optimal electrode distance (calibration value).

**Figure 17:**
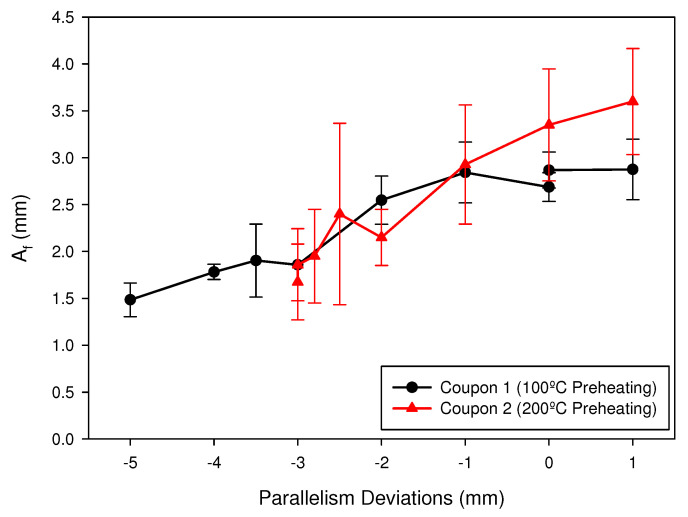
Length of the weld leg ($A_{f}$) measured for the analyzed coupons.

Figure 17 shows the variation of the length of the weld leg ($A_{f}$) as a function of the parallelism deviation for the two coupons analyzed. It can be observed that, with the preheating of 100 °C (black line), there was a significant decrease in $A_{f}$ for deviations higher, in absolute values, to −2 mm. In the case of the preheating of 200 °C (red line), this decrease was important for deviations greater, in absolute values, than −1 mm. In both cases, the interval between −1 and 1 mm around the optimum distance allowed to reach the values of $A_{f}$ required by the standard (2.7 in our case).

### 3.3. Considerations over the Header Box Manufacturing and Tube-to-Tubesheet Welding

As mentioned in the Introduction section, previous studies of this topic tend to focus on the causes of failure, operative conditions or simulations of the welding process. However, we have determined that the manufacturing of this kind of header box introduces parallelism deviation between the plugsheet (front wall) and the tubesheet (rear wall). These deviations can have a great influence on the quality and penetration of the tube-to-tubesheet welding, if they are not controlled or avoided, and an orbital GTAW equipment is used without welding height positioning and arc length control [21]. Furthermore, not only do variations in the preheating temperature have a huge influence on micrographical aspects related to material microstructure but also to weld penetration (Figure 16 and Figure 17). For those reasons, and taking into account the state of the art of this kind of equipment in the industry, the following issues should be considered to avoid these causes of failure in tube-to-tubesheet welds:

(1) Optimization of the welding sequence in the manufacturing of the header box to reduce distortion and parallelism deviation between the plugsheet and the tubesheet. As has been analyzed in this case, parallelism deviations greater than 1 mm (in absolute values) should be avoided. Obviously, a dimensional control of header boxes like the one carried out in this work could help to determine these deviations, although for productivity and economic profitability, it cannot be recommended in practical industrial procedures.
(2) Control of preheating temperature, not only due to its influence on microstructural materials aspects (embrittlement/martensite formation) but also for its influence on weld penetration, is an important issue.
(3) And specially, to implement improvements in the conventional orbital GTAW used for welding tube-to-tubesheet joints. These improvements should be addressed to the digitization of the positioned GTAW machine with the optimal electrode-joint distance in each hole and following the joint. In reference [21] we can find the different technologies that could be implemented in a conventional orbital GTAW machine to overcome the problems and consequences described in this article. This will enable to achieve optimal positioning and therefore, optimal weld penetration overcoming parallelism deviations of header box plates.

## 4. Conclusions

- A dimensional control was carried out on two header boxes of a heat exchanger in order to determine the parallelism deviations between plates (plugsheet and tubesheet). These parallelism deviations are due to the manufacturing process of the header box. They vary within −2.75 mm and 1.75 mm and may cause a bad positioning of the orbital GTAW equipment used for tube-to-tubesheet joints. Taking these values into account, the maximum error in the positioning of the welding equipment could be of around 4.5 mm.
- A coupon was designed and manufactured reproducing these deviations and for simulating the tube-to-tubesheet welding. It was determined that weld penetration decreased with increasing distance between the electrode and the joint. Negative deviations higher, in absolute values, than 1 mm result in welds do not meet the minimum requirements of manufacturing standards for this type of equipment. It was also determined that a decrease in the preheating temperature added to parallelism deviation, significantly reduced weld penetration.
- Finally, the incorporation of dimensional controls and optimization of the welding sequence during the manufacturing of the header box were discussed, and improvements in the automatic positioning automatically of this orbital GTAW equipment should be considered as the optimal solution for this kind of heat exchangers.

## Figures and Tables

**Table 1:** Chemical composition of materials [29].

SA-516 Gr. 70 (Tubesheet)		SA-210 Gr. A1 (Tube)	
Element	Composition %	Element	Composition %
Carbon, max	0.28 (50 mm thickness)	Carbon, max	0.27
Manganese	Heat analysis 0.85–1.20 Product analysis 0.79–1.30	Manganese, max	0.93
Phosphorus, max	0.025	Phosphorus, max	0.035
Sulfur, max	0.025	Sulfur, max	0.035
Silicon	Heat analysis 0.15–0.40 Product analysis 0.13–0.45	Silicon, min	0.10
Carbon Equivalent (CE)	0.5	Carbon Equivalent (CE)	0.42

## Data Availability

The data presented in this study are available on reasonable request from the corresponding author.

## Abbreviations

The following abbreviations are used in this manuscript:

GTAW	Gas Tungsten Arc Welding
TIG	Tungsten Inert Gas
MLP	Minimum leak pathThree
$A_{f}$	Length of leg
HAZ	Heat affected zone
PWHT	Post Weld Heat Treatment
CE	Carbon Equivalent
